# Association of Polygenic Risk Score With Lifetime Risk of Developing Multiple Sclerosis in a Population-Based Birth-Year Cohort

**DOI:** 10.1212/WNL.0000000000209663

**Published:** 2024-09-13

**Authors:** Floor C. Loonstra, Daniel Álvarez Sirvent, Niccoló Tesi, Henne Holstege, Eva M.M. Strijbis, Alex N. Salazar, Marc Hulsman, Sven J. Van Der Lee, Bernard Uitdehaag

**Affiliations:** From the MS Center Amsterdam (F.C.L., E.M.M.S., B.U.), Neurology, Amsterdam Neuroscience, Genomics of Neurodegenerative Diseases and Aging (D.Á.S., N.T., H.H., A.N.S., M.H., S.J.V.D.L.), Human Genetics, and Alzheimer Center Amsterdam (H.H., S.J.V.D.L.), Neurology, Vrije Universiteit Amsterdam, Amsterdam UMC location VUmc; Delft Bioinformatics Lab (N.T., H.H.), Delft University of Technology; and Amsterdam Neuroscience (H.H., S.J.V.D.L.), Neurodegeneration, the Netherlands.

## Abstract

**Background and Objectives:**

More than 200 genetic variants have been associated with multiple sclerosis (MS) susceptibility. However, it is unclear to what extent genetic factors influence lifetime risk of MS. Using a population-based birth-year cohort, we investigate the effect of genetics on lifetime risk of MS.

**Methods:**

In the Project Y study, we tracked down almost all persons with MS (pwMS) from birth year 1966 in the Netherlands. As control participants, we included non-MS participants from the Project Y cohort (born 1965–1967 in the Netherlands) and non-MS participants from the Amsterdam Dementia Cohort born between 1963 and 1969. Genetic variants associated with MS were determined in pwMS and control participants using genotyping or imputation methods. Polygenic risk scores (PRSs) based on variants and weights from the largest genetic study in MS were calculated for each participant and assigned into deciles based on the PRS distribution in the control participants. We examined the lifetime risk for each decile and the association between PRS and MS disease variables, including age at onset and time to secondary progression.

**Results:**

MS-PRS was calculated for 285 pwMS (mean age 53.0 ± 0.9 years, 72.3% female) and 267 control participants (mean age 51.8 ± 3.2 years, 58.1% female). Based on the lifetime risk estimation, we observed that 1:2,739 of the women with the lowest 30% genetic risk developed MS, whereas 1:92 of the women with the top 10% highest risk developed MS. For men, only 1:7,900 developed MS in the lowest 30% genetic risk group, compared with 1:293 men with the top 10% genetic risk. The PRS was not significantly associated with age at onset and time to secondary progression in both sexes.

**Discussion:**

Our results show that the lifetime risk of MS is strongly influenced by genetic factors. Our findings have the potential to support diagnostic certainty in individuals with suspected MS: a high PRS could strengthen a diagnosis, but especially a PRS from the lowest tail of the PRS distribution should be considered a red flag and could prevent misdiagnosing conditions that mimic MS.

## Introduction

Multiple sclerosis (MS) is a disabling neurologic disorder determined by environmental and genetic risk factors.^1^ The estimated average lifetime risk of MS worldwide is approximately 5/1,000 for women and 2/1,000 for men.^2^ Genetic factors play an important role in MS: thus far, over 200 genetic susceptibility loci and 30 major histocompatibility complex (MHC) variants have been identified through genome-wide association studies (GWASs) in European individuals.^3^ However, most genetic variants only carry a small effect, such that combining variants into polygenic risk scores (PRSs) results in modest predictions of MS development.^4,5^ Furthermore, a recent GWAS has shown that PRSs composed of risk variants do not contribute to disease severity but have a moderate effect on age at onset.^6^ It is currently unclear to what extent genetic factors influence the lifetime risk of MS.

Investigating the influence of PRSs on lifetime risk is methodologically challenging. First, each genetic locus is associated with MS with a certain effect size (odds ratio), which cannot be directly translated to lifetime risk.^7^ Second, the relatively low incidence of MS limits the use of prospective population-based cohort studies to accurately determine the lifetime risk of MS. Third, patient cohorts are influenced by selection bias and often include patients of variable ages, such that participants are exposed to different environments. To overcome these challenges, we used a population-based birth-year cohort (Project Y) and age-matched participants to investigate the association between the PRS and the lifetime risk of MS. In addition, we tested the association of the PRS in our cohort with relevant clinical outcome variables.

## Methods

### Study Participants

We included participants from 2 cohorts: persons with MS (pwMS) and control participants from the Project Y cohort and age-matched control participants from the Amsterdam Dementia Cohort (ADC). pwMS were included in the Project Y cohort who met the following criteria: (1) born in 1966 in the Netherlands; (2) currently living in the Netherlands; (3) diagnosis of MS according to the 2010^8^ or 2017^9^ McDonald criteria. Project Y was promoted across various media platforms, including radio broadcasts, social media, newspapers, and flyers and websites of MS patient associations. Neurologists specializing in MS from all hospitals in the Netherlands were approached and requested to distribute information packages to all pwMS born in 1966. In addition, outreach was made to nursing homes across the country. Using this approach, the Project Y cohort was able to identify almost *all* pwMS born in 1966 (239,611 live births) in the Netherlands.^10,11^ During the 1-day study visit between December 2017 and January 2021, data on MS disease course, including age at first symptom onset and time to secondary progression, were obtained through interviews and the review of patients' medical records.^10^ In addition to Project Y participants, we included non-MS participants from the ADC^12^ born between 1963 and 1969. The ADC comprises individuals who visit the Alzheimer Center Amsterdam and agree to the use of their clinical data for scientific purposes. To determine sex of all participants, we used self-identified sex combined with medical record review. Sex was determined at birth. The Medical Ethical Committee of the Amsterdam University Medical Center approved the Project Y and the ADC cohorts: all participants and/or their legal guardians gave written informed consent for participation in clinical and genetic studies. This study followed the STROBE reporting guidelines.

### Genotyping

All participants were genotyped on the same genotyping array (Genome Screening Array version 3): a total of 420 participants from the Project Y cohort and 1918 from ADC were included. Genetic variants were determined using standard imputation methods and by the application of established quality control methods.^13,14^ In brief, high-quality genotypes (individual call rate >99%, variant call rate >99%) were used for all participants. Significance in departure from Hardy-Weinberg equilibrium was set at *p* < 1 × 10^−6^. Participants with sex mismatches (N = 11 for ADC, N = 2 for Project Y) were excluded. Subsequently, genotypes were lifted over to GRCh38 and prepared for imputation using provided scripts (HRC-1000G-check-bim.pl), specifying TOPMED as the reference panel.^15^ This script compares variant ID, strand, and allele frequencies with the TOPMED reference panel (version r2, N = 194,512 haplotypes from N = 97,256 individuals). All variants were submitted to the TOPMED Imputation server. The server uses EAGLE (v2.4) to phase data and Minimac4 to perform genotype imputation to the reference panel (version r2). Before downstream analyses, we excluded participants with a family relation (identity-by-descent ≥0.2, N = 13 for ADC, N = 12 for Project Y),^16^ and we excluded participants of non-European ancestry (based on 1000Genomes clustering, N = 203 for ADC, N = 8 for Project Y).^17^ Finally, we restricted the analysis to participants born between 1963 and 1969.

### PRS Calculation

For PRS calculation, we considered 215 susceptibility variants (*p* < 5 × 10^−8^) from the largest MS GWAS to date, which included 47,429 pwMS and 68,374 control participants.^3^ All included variants were autosomal, with 15 of them being located in the MHC region in chromosome 6. MHC variants were imputed using software HIBAG^18^ and the prediction model specific to the genotyping platform Illumina Infinium Global Screen Array version 2.0 for populations of European ancestry. The quality control threshold for posterior probability of variants was set at >0.5, as recommended by the authors. PRSs were calculated as the sum of susceptibility variants per individual, weighted by their effect sizes as available in the MS GWAS summary statistics. Effect sizes were calculated as the log of the odds ratios of the variants. PRSs were scaled by means of Z-score standardization.

### Lifetime Risk Calculation

The PRSs of the pwMS were assigned into deciles of genetic risk based on the PRS distribution of the control participants. For the lifetime risk calculation, we used a simulated population of 100,000 participants per decile, simulating the full cohort of live births in 1966. This simulated population was based on male-specific and female-specific birth rates and the age-specific lifetime MS risk estimation from the Project Y cohort. We assumed the distribution of the PRS in control participants to be comparable with the distribution in the entire population. Each decile then contains the same proportion of pwMS as the respective decile in our study cohort. The accumulated lifetime risk per decile is calculated separately for men and women using the following formula: sex-specific lifetime risk estimates of the 1966 cohort × proportion of pwMS in respective decile/100,000.

### Associations Between PRS and Clinical Variables

We tested the association between PRSs and age at onset of MS, age of MS progression (time to secondary progression from birth), and time to secondary progression (also reported as disease duration at secondary progression or time to secondary progression from disease onset). All associations were stratified by sex. For the associations, we used Cox proportional hazard regression models, adjusting for sex (when not stratified) and population effect (5 genetic principal components). We censored time to secondary progression at the time of visit in patients lacking information for the age at secondary MS progression. Cox proportional hazard assumption was positively tested in all models, as well as linearity assumption for continuous predictors, homoscedasticity of outliers, and multicollinearity (by Variance Inflation Factor testing). In addition, we performed testing per each PRS decile. Because the number of patients with MS was small in PRS deciles 1–4, we tested only PRS deciles 5–10.

Bioinformatics analysis was performed using custom scripts in the R programming language.^19^

### Data Availability

Anonymized data not published within this article will be made available on reasonable request from any qualified investigator to the corresponding author (B.M.J.U.).

## Results

### Cohort Description and Lifetime Risk Estimation

We identified 452 pwMS in the 1966 cohort (73% female), which translates to a lifetime risk estimate of 282 per 100,000 in women (329/116,745) and 100 per 100,000 in men (123/122,866).^10,11^ The PRS for MS was calculated in 285 pwMS from the Project Y cohort and 267 non-MS control participants from both the Project Y cohort and ADC. As shown in Table 1, the mean age within the pwMS group was 53.0 years (SD = 0.9), with 72.3% being female. The mean age of the control participants was 51.8 years (SD = 3.2), with 58.1% being female. The mean age at onset for the pwMS was 36.6 years (SD = 9.30) (Table 2). 59.3% pwMS had relapsing-remitting MS (n = 169), 27.7% secondary progressive MS (n = 79), and 13.0% had primary progressive MS (n = 37) (Table 2).

**Table 1:** Characteristics of the Study Cohort

Group	N	Age, mean (SD)	Male, n (%)	Female, n (%)
pwMS	285	52.98 (0.9)	79 (27.7)	206 (72.3)
Non-MS	267	51.8 (3.2)	113 (41.9)	154 (58.1)

Abbreviations: MS = multiple sclerosis; pwMS = persons with MS.

**Table 2:** Characteristics of the pwMS Group: Subtypes, Sex, and Age at Onset

MS type	N (%)	Age at onset, mean (SD)	Male, n (%)	Female, n (%)
MS all	285	36.55 (9.30)	79 (27.7)	206 (72.3)
RRMS	169	37.20 (9.18)	30 (22.3)	134 (81.7)
SPMS	79	31.88 (8.01)	31 (39.3)	48 (60.7)
PPMS	37	42.84 (7.26)	18 (48.6)	19 (51.4)

Abbreviations: MS = multiple sclerosis; PPMS = primary progressive MS; pwMS = persons with MS; RRMS = relapsing-remitting MS; SPMS = secondary progressive MS.

### PRS Associates With Risk and Lifetime Risk of MS

**Figure 1:**
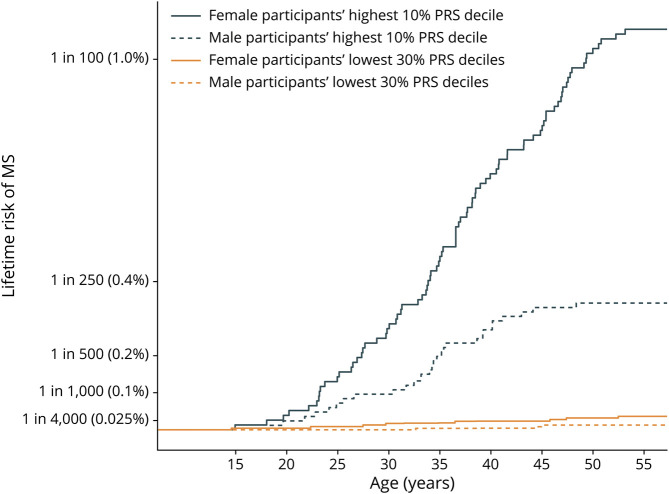
Lifetime Risk for the Lowest 30% and Highest 10% PRS Deciles by Age at Onset, Based on the Sex-Specific Lifetime Risk Estimate of the 1966 Birth Cohort PRS = polygenic risk score.

**Figure 2:**
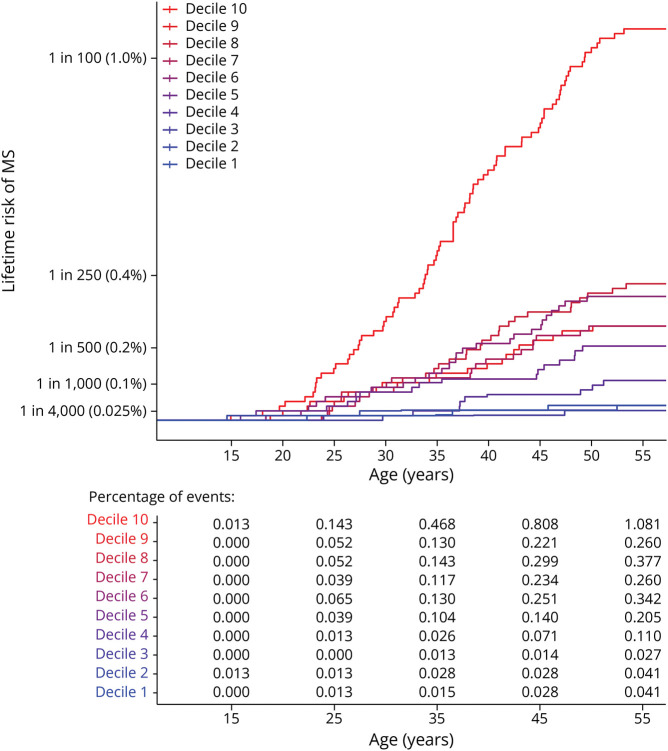
Lifetime Risk for 10 PRS Deciles in Women, by Age at Onset, Based on the Female-Specific Lifetime Risk Estimate of the 1966 Birth Cohort Risk table shows percentage of events per time point. MS = multiple sclerosis; PRS = polygenic risk score.

**Figure 3:**
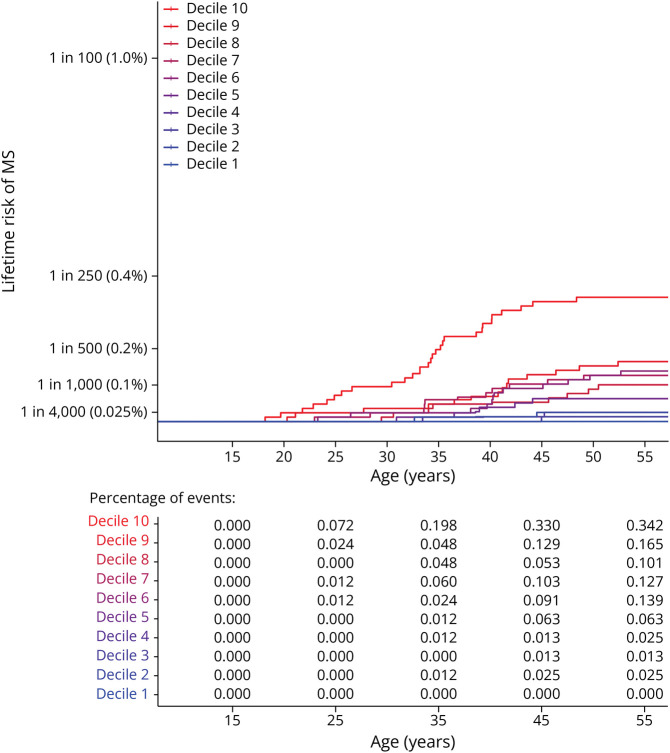
Lifetime Risk for 10 PRS Deciles in Men, by Age at Onset, Based on the Male-Specific Lifetime Risk Estimate of the 1966 Birth Cohort Risk table shows percentage of events per time point. MS = multiple sclerosis; PRS = polygenic risk score.

The PRS for MS was significantly associated with risk of developing MS (*p* = 1.07e-15, β = 0.91). Men and women with similar genetic risk were differentially affected with MS (Table 3). We simulated populations of birth year 1966, by populating equal deciles with same proportions of pwMS per decile as in our study cohort. We observed that 1:92 (1.1%) of the women with the top 10% highest risk developed MS (108/10,000), whereas only 1:2,779 of the women with the lowest 30% genetic risk developed MS (3.6/10,000) (Figure 1). For men, 1:293 (0.34%) with the top 10% genetic risk (34/10,000) developed MS, compared with 1:7,900 men in the lowest 30% genetic risk group (1.3/10,000). The relative risk (RR) of the top 10% risk vs the lowest 30% risk was comparable between women (RR = 29.4) and men (RR = 26). Figures 2 and 3 show the lifetime risk for all PRS deciles in women and men, respectively. Risk tables with percentage of events per time point are shown.

**Table 3:** Lifetime Risk for Each PRS Decile Based on the Sex-Specific Lifetime Risk Estimate of the 1966 Birth Cohort

PRS decile	Control participants, N	Female pwMS, N (%)	Lifetime risk, female pwMS	Age at onset, female pwMS, median (IQR)	Male pwMS, N (%)	Lifetime risk, male pwMS	Age at onset, male pwMS, median (IQR)
1	27 (F13, M14)	3 (2)	1 in 2,435	36.48 (11.72)	0 (0)	0	NA
2	27 (F16, M11)	3 (2)	1 in 2,345	27.47 (18.95)	2 (3)	1 in 3,950	38.57 (5.94)
3	27 (F18, M9)	2 (1)	1 in 3,652	38.55 (8.84)	1 (1)	1 in 7,900	44.96 (0)
4	27 (F16 M11)	8 (4)	1 in 913	37.45 (13.18)	2 (3)	1 in 3,950	39.33 (5.91)
5	27 (F15 M12)	15 (7)	1 in 487	33.25 (19.43)	5 (6)	1 in 1,580	38.93 (5.9)
6	27 (F17, M10)	25 (12)	1 in 292	36.85 (16.5)	11 (14)	1 in 718	40.25 (7.44)
7	27 (F12 M15)	22 (11)	1 in 332	38.28 (15.29)	10 (13)	1 in 790	36.61 (7.88)
8	26 (F13, M13)	30 (15)	1 in 243	37.86 (11.42)	8 (10)	1 in 987	40.03 (14.83)
9	26 (F18, M8)	19 (9)	1 in 384	37.95 (16.56)	13 (16)	1 in 608	41.16 (9.57)
10	26 (F16, M10)	79 (38)	1 in 92	37.01 (15.33)	27 (34)	1 in 293	34.37 (13.17)

Abbreviations: IQR = interquartile range; MS = multiple sclerosis; PRS = polygenic risk score; pwMS = persons with MS.

For both male and female pwMS, no difference in age at onset was found per decile.

### PRS Does Not Associate With Other Disease Variables

Next, we tested the association between the PRS and MS disease outcome variables. The results for the sex-stratified tests are summarized in Table 4; results for decile-stratified tests are provided in eTable 1. After false discovery rate correction using the Benjamini-Hochberg method, none of the analyses yielded statistically significant results: PRS was not significantly associated by means of Cox models with time to secondary progression (*p* = 0.84, hazard ratio [HR] = 0.93), age at MS progression (*p* = 0.981, HR = 0.98), or age at onset of MS (*p* = 0.67, HR = 1.12). Stratification per sex or PRS deciles yielded similar results.

**Table 4:** Association Between PRS and Clinical Variables, Stratified by Sex

Outcome	HR (95% CI)	SE	*p* Value	*p* Value adjusted^a^	N
Age at onset	1.12 (0.97–1.28)	0.081	0.148	0.668	282
Age at onset F	1.01 (0.82–1.20)	0.096	0.913	0.981	203
Age at onset M	1.48 (1.17–1.79)	0.157	0.013	0.116	79
Age of progression	0.98 (0.74–1.23)	0.124	0.96	0.981	276
Age of progression F	0.90 (0.57–1.23)	0.168	0.542	0.84	200
Age of progression M	1.12 (0.75–1.49)	0.189	0.551	0.84	76
Time to progression	0.93 (0.69–1.17)	0.124	0.56	0.84	276
Time to progression F	1.00 (0.69–1.32)	0.161	0.981	0.981	200
Time to progression M	0.84 (0.42–1.27)	0.219	0.441	0.84	76

Abbreviations: F = female; HR = hazard ratio; M = male.

a Benjamini-Hochberg corrected *p* value.

## Discussion

In a unique cohort of pwMS and control participants, we show that genetic factors have a profound influence on the lifetime risk of MS in men and women. We estimated lifetime risk per PRS decile, using a population-based cohort with the same year of birth. Although the lifetime risk of MS in those with the highest 10% genetic risk is >1% in women and 0.34% in men, the lifetime risk in those with the lowest 30% genetic risk is negligible.

We report no association in our study cohort between PRSs and age at first symptom onset and time to secondary progression. This finding is in line with the latest MS severity GWAS,^6^ which reports only a moderate association between PRSs and age at onset. This GWAS suggests that the genetic architectures for risk and severity of the disease might differ in MS, supported by the independence of the severity-associated genetic variants from previously identified risk genetic variants.^6^

A strength of our study is that we used a cohort of pwMS that was not included in the MS-GWAS, thereby avoiding circular fallacy. Furthermore, the cohort is a close-to-complete sample of Dutch pwMS born in 1966. We conducted an extensive search within MS registries throughout the country and also engaged in recruitment beyond the hospital setting. In addition, we introduced 3 modalities for data acquisition (hospital visit, home visit, or telephone interview). The population-based nature of this cohort is confirmed by the inclusion of a substantial number of pwMS from assisted care facilities, as well as pwMS with an EDSS score higher than 8.0 and a significant number of recently diagnosed pwMS. However, the estimates are representative of the 1966 Dutch cohort and may not translate to other birth years or populations.

A limitation of our study is that results are not generalizable to non-European populations. The genetic variants included in the PRS were taken from the MS GWAS, which included mainly participants of European ancestry. Genetic factors that affect risk of MS might differ per population, in frequency and effect size. Thus, implementation of a mainly European-based PRS in populations of different ancestries might yield different results. In addition, our PRS calculation was based on the MS GWAS,^3^ but the genetic complexity of MS susceptibility remains incompletely understood, which could have affected our lifetime risk estimations.

Another factor that could have affected the estimates was that competing risk of death was not taken into account. We considered the competing risk of death to be negligible, given that the number of deaths during the study period was insubstantial compared with disease onset. The percentage of participants who died before the initiation of Project Y was comparable in patients with MS (17 of 452, 3.7%) and birth cohort participants (4.9% of men and 3.6% of women born in 1966, age from 15 to 56 years).

Our results have the potential to support diagnostic certainty in patients with suspected MS. A high PRS could strengthen a diagnosis. It is important to note that a PRS from the lowest tail of the PRS distribution could prevent misdiagnosing conditions that mimic MS and should be considered a red flag.

## Appendix Authors

**Table:**

Name	Location	Contribution
Floor C. Loonstra, MD	MS Center Amsterdam, Neurology, Vrije Universiteit Amsterdam, Amsterdam Neuroscience, Amsterdam UMC location VUmc, the Netherlands	Drafting/revision of the manuscript for content, including medical writing for content; major role in the acquisition of data; study concept or design; analysis or interpretation of data
Daniel Álvarez Sirvent, MSc	Genomics of Neurodegenerative Diseases and Aging, Human Genetics, Vrije Universiteit Amsterdam, Amsterdam UMC, location VUmc, the Netherlands	Drafting/revision of the manuscript for content, including medical writing for content; analysis or interpretation of data
Niccoló Tesi, PhD	Genomics of Neurodegenerative Diseases and Aging, Human Genetics, Vrije Universiteit Amsterdam, Amsterdam UMC, location VUmc; Delft Bioinformatics Lab, Delft University of Technology, the Netherlands	Drafting/revision of the manuscript for content, including medical writing for content; analysis or interpretation of data
Henne Holstege, PhD	Genomics of Neurodegenerative Diseases and Aging, Human Genetics, and Alzheimer Center Amsterdam, Neurology, Vrije Universiteit Amsterdam, Amsterdam UMC location VUmc; Delft Bioinformatics Lab, Delft University of Technology; Amsterdam Neuroscience, Neurodegeneration, the Netherlands	Drafting/revision of the manuscript for content, including medical writing for content; analysis or interpretation of data
Eva M.M. Strijbis, MD, PhD	MS Center Amsterdam, Neurology, Vrije Universiteit Amsterdam, Amsterdam Neuroscience, Amsterdam UMC location VUmc, the Netherlands	Drafting/revision of the manuscript for content, including medical writing for content
Alex N. Salazar, PhD	Genomics of Neurodegenerative Diseases and Aging, Human Genetics, Vrije Universiteit Amsterdam, Amsterdam UMC, location VUmc, the Netherlands	Analysis or interpretation of data
Marc Hulsman, PhD	Genomics of Neurodegenerative Diseases and Aging, Human Genetics, Vrije Universiteit Amsterdam, Amsterdam UMC, location VUmc, the Netherlands	Analysis or interpretation of data
Sven J. Van Der Lee, MD, PhD	Genomics of Neurodegenerative Diseases and Aging, Human Genetics, and Alzheimer Center Amsterdam, Neurology, Vrije Universiteit Amsterdam, Amsterdam UMC location VUmc; Amsterdam Neuroscience, Neurodegeneration, the Netherlands	Drafting/revision of the manuscript for content, including medical writing for content; major role in the acquisition of data; study concept or design; analysis or interpretation of data
Bernard Uitdehaag, MD, PhD	MS Center Amsterdam, Neurology, Vrije Universiteit Amsterdam, Amsterdam Neuroscience, Amsterdam UMC location VUmc, the Netherlands	Drafting/revision of the manuscript for content, including medical writing for content; major role in the acquisition of data; study concept or design; analysis or interpretation of data

## Glossary

ADC: Amsterdam Dementia Cohort
GWAS: genome-wide association study
HR: hazard ratio
MHC: major histocompatibility complex
MS: multiple sclerosis
PRS: polygenic risk score
pwMS: persons with MS
RR: relative risk
